# Innovations in topical epidermolysis bullosa treatment: integrating advanced dressings, bioactive therapies and tissue-engineered skin

**DOI:** 10.1007/s40199-026-00601-5

**Published:** 2026-04-03

**Authors:** Mandeep Kaur Marwah, Kirandeep Kaur, Shakil Ahmad, Harmony C. K. Cheema

**Affiliations:** Aston Medical School, Aston University, Birmingham, UK

## Abstract

**Objectives:**

Epidermolysis Bullosa (EB) is a rare genetic disorder characterised by extreme skin fragility, chronic wounds, and high morbidity. Current management is largely supportive, highlighting an urgent need for innovative therapeutic strategies. This review synthesises recent advances in topical therapies, including wound dressings, tissue-engineered skin, and molecular therapies, and evaluates the potential of these approaches to improve clinical outcomes and bridge the gap toward disease-modifying interventions.

**Evidence acquisition:**

Key developments in EB pathophysiology, advanced wound-care technologies, topical molecular therapies, and cell- or tissue-based interventions were examined through critical appraisal of relevant preclinical and clinical studies, with emphasis on translational potential and practical application.

**Results:**

Next-generation dressings incorporating hydrogels, extracellular matrix-mimetic scaffolds, nanoparticles, and smart-responsive systems demonstrate enhanced protection, moisture balance, and localised therapeutic delivery, supporting wound healing while minimising trauma. Topical molecular therapies—including gene therapy, repurposed drugs, and bioactive natural compounds—show proof-of-concept for symptomatic relief and potential disease modification. Cell- and tissue-based approaches, such as ex vivo gene-corrected keratinocyte grafts and cultured skin substitutes, offer durable regeneration but remain limited by cost and accessibility. Integration of these strategies, combined with patient-centred design and co-development, holds promise for improving functional outcomes and quality of life in EB.

**Conclusion:**

Advanced wound dressings, bioactive topical therapies, and tissue-engineered constructs represent complementary and translationally relevant approaches to EB care. Optimising the synergy between material-based and molecular strategies, alongside standardised clinical endpoints and long-term safety evaluation, is essential for bridging supportive care and future disease-modifying therapies, ultimately improving outcomes for patients with EB.

## Introduction

Epidermolysis Bullosa (EB) is a rare, genetically heterogeneous group of inherited disorders affecting the skin and other epithelial tissues, characterised by extreme fragility of these surfaces and resulting in blistering and erosions in response to minimal mechanical trauma [1, 2]. Manifestations can range from mild blistering in localised areas to severe, widespread lesions affecting multiple organ systems [3]. EB is most readily visible on the skin, however, any organ lined by epithelium can be affected, and extracutaneous manifestations often include involvement of the eye, airway, and gastrointestinal or genitourinary tracts [4]. The disease typically presents at birth or in early childhood and is caused by mutations in genes encoding structural proteins critical for maintaining skin integrity, including keratins, laminins, and collagens [1]. These molecular defects compromise the dermoepidermal junction, leading to the hallmark clinical features of EB and a host of chronic complications [5].

EB is rare, with an estimated incidence of 1 in 25,000–50,000 live births worldwide, however its impact is profound [6, 7]. In addition to the immediate skin manifestations, patients experience chronic wounds, recurrent infections, anaemia, nutritional deficiencies, and a significantly increased risk of malignancy [8]. The disease imposes substantial psychosocial and economic burdens, as lifelong intensive care, frequent hospital visits, and specialised support are required to manage its complications [8].

Despite advances in supportive care, EB remains incurable, and existing therapies are largely symptomatic, focusing on wound management, pain control, infection prevention, and nutritional support [9]. These measures, while essential, do not address the underlying genetic defects, nor do they prevent long-term complications such as scarring and cancer. Consequently, there is an urgent need for novel therapies that target the molecular and cellular basis of the disease. Emerging therapeutic strategies such as gene replacement and editing, protein replacement, and cell-based therapies offer the possibility of correcting the underlying molecular defects and achieving durable or potentially curative outcomes [2]. While these approaches represent an important long-term objective, substantial scientific, regulatory and economic challenges currently constrain broad availability and routine clinical implementation. In the meantime, there is growing interest in next-generation topical treatments that move beyond passive protection to incorporate bioactive, regenerative, antimicrobial or smart-responsive functions. As skin fragility and chronic wounds remain the most persistent and burdensome clinical features across all EB subtypes, this review uniquely integrates innovations in dressing materials, tissue-engineered skin, and molecular therapies, representing a highly relevant and pragmatic therapeutic pathway with potential to deliver near-term improvements in healing, comfort and quality of life. By critically evaluating the advantages, limitations, and translational relevance of each approach, we provide a focused perspective on emerging topical strategies that can bridge the gap between current symptomatic care and future disease-modifying therapies.

## Pathophysiology of EB

EB arises from inherited mutations that impair the structural stability of the skin. It is genetically heterogeneous, with over twenty causative genes identified across the principal EB subtypes [2]. These mutations affect proteins responsible for cytoskeletal anchorage, basement membrane integrity, and dermal–epidermal adhesion, resulting in marked skin fragility and blister formation [10]. Importantly, the specific mutation determines the precise plane of the mechanical failure within the skin. Defects in keratins (KRT5, KRT14) compromise the cytoskeletal resilience of basal keratinocytes, while mutations affecting laminins (LAMA3, LAMB3, LAMC2), type XVII collagen (COL17A1), integrins (ITGA6, ITGB4), or type VII collagen (COL7A1) destabilise adhesion at or below the basement membrane zone [11]. This level-specific fragility imposes fundamental design constraints for wound dressings in EB, as even minimal adhesive force, shear, or peel stress applied across these interfaces can precipitate blistering and tissue loss underscoring the need for dressings engineered to minimise adhesion and mechanical stress.

**Fig. 1:**
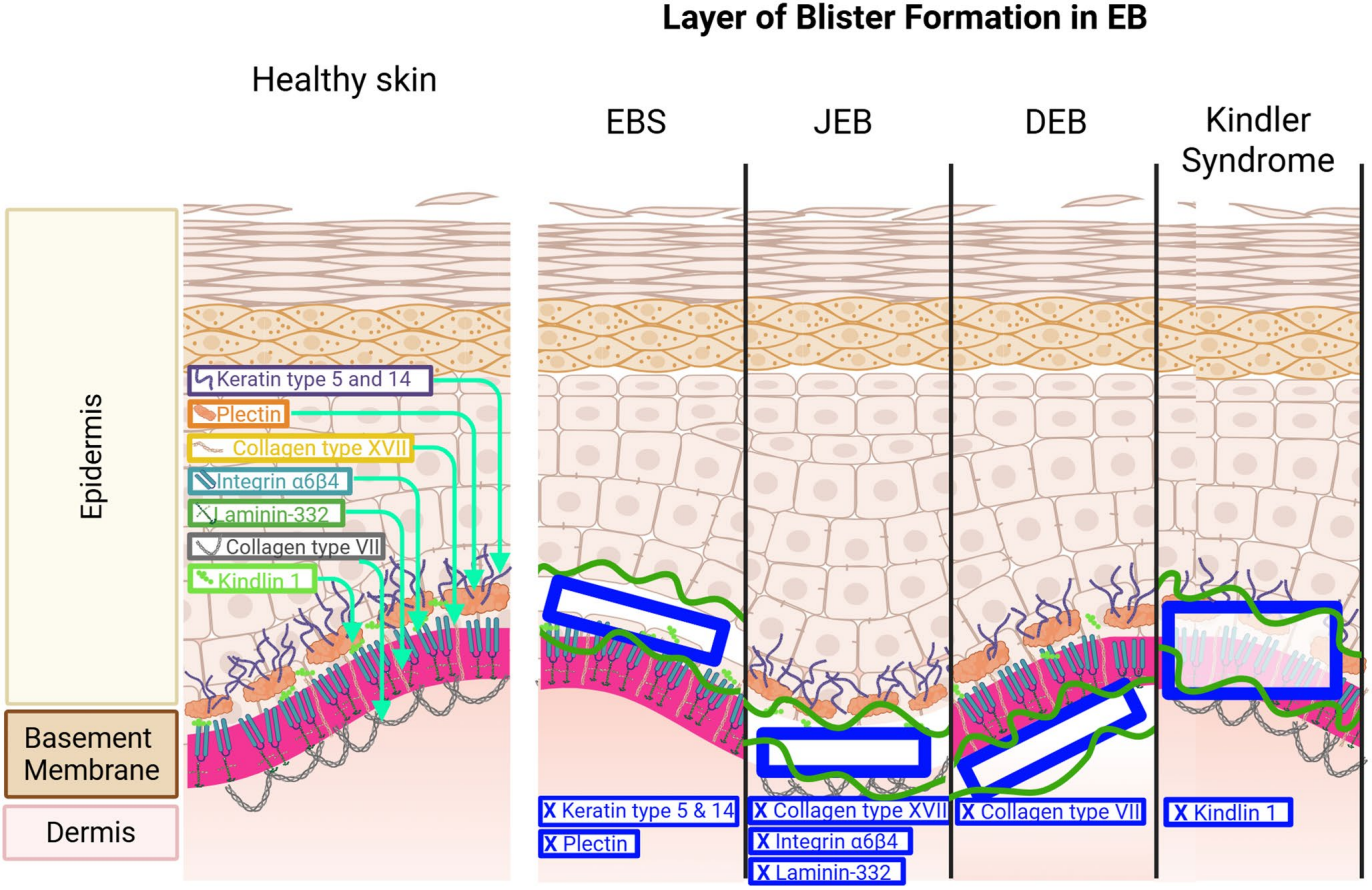
Classification and molecular basis of epidermolysis bullosa. Epidermolysis bullosa comprises four major subtypes defined by the level of skin cleavage and the affected structural proteins. Epidermolysis bullosa simplex (EBS) results from mutations in keratin 5 or 14, causing basal keratinocyte fragility and intraepidermal blistering. Junctional EB (JEB) arises from defects in laminin-332, type XVII collagen, or integrins, leading to separation within the lamina lucida. Dystrophic EB (DEB) is caused by loss of type VII collagen anchoring fibrils, resulting in sublamina densa blistering. Kindler syndrome is associated with defects in Kindlin 1, leading to disrupted focal adhesions and variable cleavage planes. Created in BioRender. Marwah, M. (2026) https://BioRender.com/49z7o7h

EB is conventionally classified into four major subtypes based on the level of skin cleavage and the affected protein: EB simplex, junctional EB, dystrophic EB, and Kindler syndrome [11] (Fig. 1). In EB simplex, mutations in keratin 5 or 14 destabilise the keratin intermediate filament network within basal keratinocytes, rendering them highly susceptible to mechanical stress and leading to intraepidermal blistering which can range from mild, localised lesions to more severe, widespread blistering [12]. Junctional EB is generally more severe, with blistering at the lamina lucida due to defects in laminins, type XVII collagen, or integrins which impairs the anchoring of basal keratinocytes to the basement membrane, causing separation within the lamina lucida and can be life-threatening in infancy [11]. Dystrophic EB is characterised by defective type VII collagen, a major component of anchoring fibrils that secure the dermis to the basement membrane; its loss results in sublamina densa blistering, scarring, dermal fibrosis, pseudosyndactyly, and a high risk of aggressive squamous cell carcinoma [13, 14]. Kindler syndrome is a rare variant arising from mutations in FERMT1 which encodes the kindlin-1 protein, disrupts focal adhesion dynamics, leading to variable cleavage planes, photosensitivity, and progressive skin atrophy [15–17].

In addition to mechanical blistering, chronic wounds and impaired healing are prominent features in severe EB subtypes, such as recessive dystrophic EB and junctional EB [18, 19]. Chronic wounds in these EB subtypes exhibit persistent inflammation, altered keratinocyte migration, and delayed re-epithelialisation [18]. Repeated tissue injury and infection lead to continuous cycles of inflammation and repair, promoting fibrosis and scarring, particularly in dystrophic EB. The chronic inflammatory environment also contributes to the high risk of aggressive cutaneous squamous cell carcinoma, one of the most serious complications of severe EB subtypes [20].

The pathophysiological consequences of EB extend beyond the skin. Patients frequently experience systemic complications, including chronic anaemia from ongoing blood loss, malnutrition due to painful oral and oesophageal lesions, and recurrent infections arising from disrupted epithelial barriers [9, 21]. In dystrophic EB, oesophageal strictures, corneal erosions, and renal involvement may occur, while growth failure and delayed puberty are common in severe forms [4, 22]. The cumulative burden of these complications contributes to significant morbidity and reduced life expectancy, particularly in junctional and severe dystrophic variants.

## Current standard of care

The management of EB remains largely supportive and symptomatic, aiming to minimise trauma, promote wound healing, control pain and infection, and optimise nutrition [9]. Advanced wound care strategies, including specialised dressings and skin grafts are often required to support healing in chronic or complex wounds [23]. Surgery may be required in severe cases to address complications such as contractures, pseudosyndactyly or strictures, although healing is often slow and recurrence is common [24]. Given the absence of a curative therapy, care is focused on improving quality of life and preventing secondary complications. Multidisciplinary management involving dermatologists, surgeons, nutritionists, pain specialists, and psychologists is essential to address the complex clinical manifestations of the disease.

Wound care forms the cornerstone of EB management. A key principle guiding contemporary wound management is the concept of moist wound healing, which supports re-epithelialisation by preventing tissue dehydration, limiting keratinocyte apoptosis, and promoting optimal cellular and growth factor activity [25]. Modern dressings are designed aim to provide non-adherent protection, manage exudate, and reduce pain, yet there is limited high-quality evidence demonstrating the superiority of specific products [26]. Unlike typical acute or chronic wounds, EB wounds arise from minimal friction, recur continuously, and occur against a background of extreme skin fragility, thus dressings must minimise shear, avoid adhesive trauma, conform to delicate contours, and permit atraumatic removal - requirements many existing products struggle to meet [26]. Fixation materials including tapes, and bandages, are poorly suited to fragile EB skin, resulting in uncomfortable, unstable dressing systems [27].

Pain management and itch management is a major component of EB care due to the constant presence of open wounds, repeated dressing changes and other related complications [28]. Systemic analgesics, are commonly used alongside topical anaesthetics [29]. Neuropathic pain is increasingly recognised as a significant contributor to the disease burden and may require adjuvant therapies such as gabapentinoids or tricyclic antidepressants [30, 31]. Psychological support is equally important, as chronic pain, disfigurement, and social isolation can lead to anxiety, depression, and reduced quality of life [29].

Chronic wounds serve as a nidus for bacterial colonisation. Management is primarily preventative and topical with the aim of reducing microbial burden [27, 32, 33]. Topical antimicrobial therapy is commonly used for short, targeted courses in the presence of clinically infected or heavily colonised wounds. Agents such as mupirocin or fusidic acid may be applied for localised Staphylococcus aureus infection, while antiseptic-based preparations such as polyhexanide, dilute povidone–iodine, chlorhexidine, or hypochlorous acid solutions, are frequently preferred for routine wound cleansing or broader antimicrobial coverage [27, 32]. Prolonged use of topical antibiotics is generally avoided in EB, as chronic exposure has been associated with the emergence of resistant organisms [32]. Consequently, topical antimicrobial strategies are typically combined with meticulous wound hygiene and atraumatic dressings to control bioburden while preserving fragile tissue [9]. Systemic antibiotics are reserved for clinically significant infection, such as spreading cellulitis, systemic symptoms, or suspected deep tissue involvement, rather than routine wound colonisation [33].

Nutritional support is essential for promoting wound healing and growth, particularly in children. Oesophageal blistering, oral erosions, and dental anomalies often impair feeding, leading to malnutrition and anaemia [1, 34, 35]. High-protein, high-calorie diets, vitamin and mineral supplementation, and in severe cases, gastrostomy feeding are recommended to meet metabolic demands [36].

Despite these measures, current management remains largely palliative, providing symptomatic relief rather than addressing the underlying disease [37]. Chronic wounds, progressive scarring, and the high incidence of aggressive squamous cell carcinoma continue to pose major clinical challenges, while the intensive, lifelong care required imposes a substantial physical, emotional, and financial burden on patients and their families [38, 39]. Consequently, there is growing interest in next-generation topical treatments that move beyond passive protection to incorporate bioactive, regenerative, or smart-responsive functions to enhance quality of life.

## Evidence acquisition

This review is based on an expert narrative synthesis of the current literature relating to wound care, biomaterial-based dressings, topical molecular therapies, and cell- and tissue-based interventions for Epidermolysis Bullosa. Literature searches were conducted using PubMed and Web of Science, supplemented by ClinicalTrials.gov and regulatory agency communications where relevant. Publications from January 1990 to 2025 were considered. Relevant preclinical studies, clinical trials, regulatory approvals, and translational research reports were identified through targeted evaluation of peer-reviewed publications, clinical trial records, and authoritative regulatory communications. Emphasis was placed on therapies with demonstrated relevance to EB pathophysiology, wound healing, or skin integrity, as well as emerging technologies with clear translational potential. Evidence was critically appraised with consideration of study design, disease subtype, mechanistic rationale, and clinical applicability, rather than through formal systematic review or meta-analytic methodology.

## Next-generation topical treatments and translational approaches in EB

### Positioning topical treatments within the EB therapeutic landscape

**Fig. 2:**
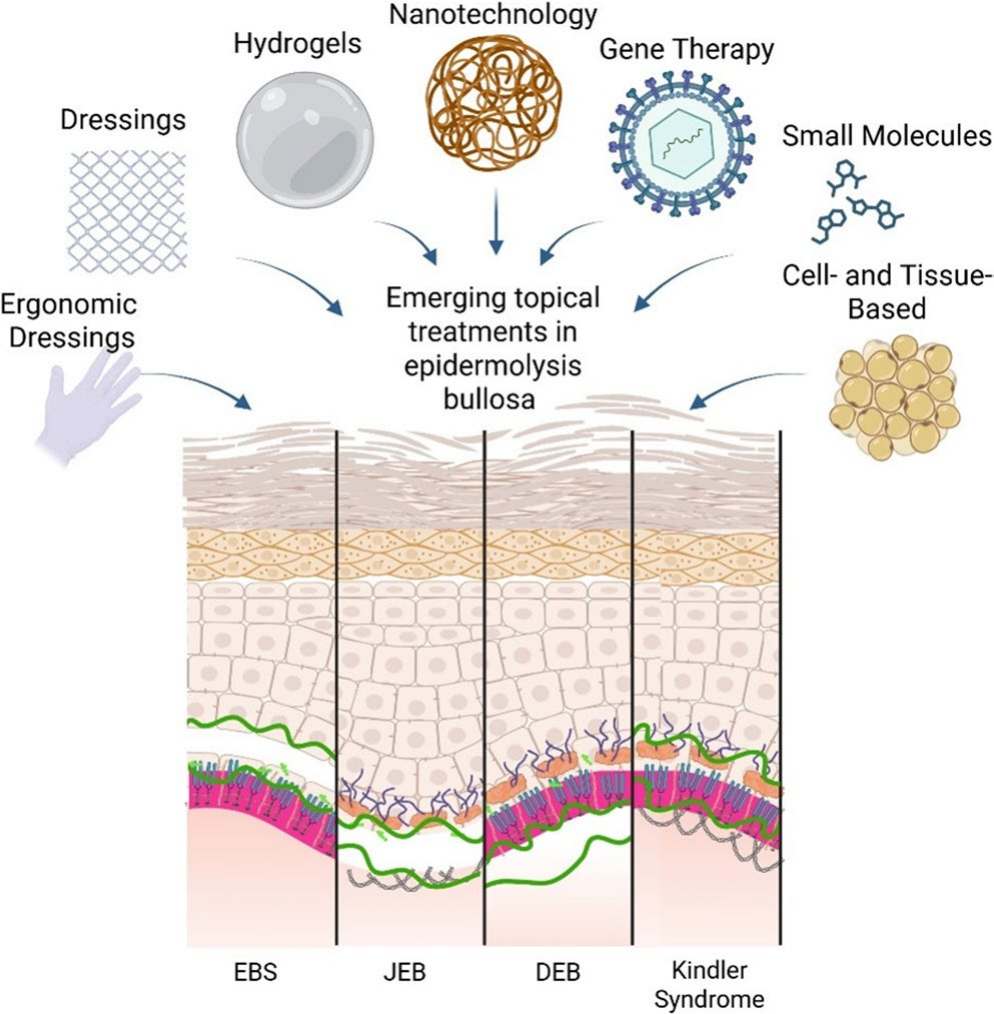
Emerging topical therapeutic strategies for epidermolysis bullosa. Illustration of selected topical and skin-targeted treatment approaches currently under investigation for epidermolysis bullosa, including protective glove and conventional dressings, bioactive hydrogels, nanoparticle-based delivery systems, gene-based therapies, small-molecule mechanistic interventions, and cell- and tissue-based strategies. Created in BioRender. Marwah, M. (2026) https://BioRender.com/49z7o7h5.2 Dressing-Based Approaches

Emerging disease-modifying strategies in EB, including gene replacement and editing, protein replacement, and cell-based therapies, offer the potential to correct underlying molecular defects and restore skin integrity at a mechanistic level [40]. While these approaches represent a promising long-term horizon clinical implementation remains largely experimental and is limited by technical, regulatory, manufacturing, and economic challenges [40, 41]. As a result, most patients continue to rely on supportive care for daily management of chronic wounds, pain, and associated complications [9]. In this context, advanced wound dressings and topical interventions have emerged as practical and translationally relevant therapeutic strategies (Table 1; Fig. 2). By combining protective, regenerative, antimicrobial, and stimuli-responsive properties, these technologies aim to address the most immediate and universal clinical burden in EB - fragile, non-healing skin, while bridging the gap between current palliative strategies and future disease-modifying interventions. Importantly, the formulation or delivery vehicle itself, such as hydrogels, can provide mechanical support, maintain a moist and protective wound environment, and modulate local tissue responses, thereby enhancing the efficacy of the encapsulated therapeutic agent beyond simple drug delivery [42, 43].

**Table 1:** Advanced Dressings, Tissue-Engineered Constructs, and Molecular Therapies for EB

Therapy	Mechanism of Action	EB Application & Benefits	Limitations	Development Stage	Reference
Knitted/Seamless Dressing Glove	Ergonomic textile dressing; low-adherence silicone reduces shear; antimicrobial coating	Hand wounds in RDEB; reduces friction, maceration, pain; conforms to web spaces	Does not actively promote healing; requires correct sizing and adherence; limited durability in heavily exudative wounds	Developmental/ early clinical use	[44]
Biobrane	Silicone–nylon mesh with porcine collagen; temporary dermal substitute promoting epithelialisation	Hand contractures, pseudosyndactyly; reduces iatrogenic trauma	Xenogeneic component; infection risk; not disease-modifying; limited EB-specific evidence	Clinical / adjunctive use	[45]
Hydrogels (chitosan, alginate, polyhexanide)	ECM-mimetic, moisture-retentive; can deliver antimicrobials or anti-inflammatories	Supports re-epithelialisation, cell migration, antimicrobial effects	Limited mechanical strength; frequent re-application; bioactivity depends on formulation	Preclinical / early clinical	[45–51] [46–51].
Dendritic / Responsive Hydrogels	Smart polymer networks that dissolve or release payloads in response to environmental cues (pH, temperature, enzymes)	Minimises trauma during removal; maintains moist wound environment	Largely preclinical; manufacturing complexity; limited long-term safety/durability	Preclinical	[52, 53]
Nanoparticle-based systems	Nano-carriers enhance active molecule stability, solubility, sustained delivery; intracellular uptake possible	Prolonged delivery of active molecule; can be incorporated into dressings or hydrogels	Preclinical data; limited EB-specific clinical data; potential tissue accumulation; scalability challenges	Preclinical	[54–57], 58, 59, 60, 61, 62]
Vyjuvek (B-VEC)	HSV-1 vector delivers COL7A1 gene to restore anchoring fibrils	DEB; accelerates wound healing, restores collagen VII	Requires repeated application; high cost; only for COL7-deficient DEB	FDA-approved	[63–66]
Filsuvez - Birch bark triterpene gel;	Modulates inflammation, promotes keratinocyte migration	Junctional EB and DEB; accelerates early wound closure	Does not correct genetic defect; wound-level benefit only	FDA-approved	[67, 68]
INM-755- Topical cannabinol	Modulates neuronal and keratinocyte activity	Symptom relief, pruritus, wound care across EB subtypes	Limited peer-reviewed evidence; small sample size; short-term	Early clinical / Phase II	[69–74].
Topical Ropivacaine	Local anaesthetic; inhibits sodium channels, reduces TRPV1 activity	Pain relief during dressing changes	Symptomatic only; evidence limited; short duration	Early clinical / Phase II	[75–77]
Diacerein	Anti-inflammatory anthraquinone; modulates IL-1 signalling	EBS; reduction in blistering	Trial heterogeneity; endpoint challenges; not universally effective	Clinical / Phase II/III	[78–80].
BM-3103 (TolaSure)	mTOR/autophagy modulation; stabilises keratin intermediate filaments	Severe EBS; reduces keratin aggregation	Early phase; peer-reviewed efficacy data not yet available	Preclinical / Phase I/IIa	[81]
Gentamicin	Nonsense mutation read-through; restores COL7 expression	RDEB with COL7A1 nonsense mutations	Limited data; optimal dosing/durability unknown	Early clinical / pilot studies	[82, 83]
Sirolimus	mTOR inhibitor; reduces keratinocyte hyperproliferation, inflammation	Plantar EBS; blister reduction, improved mobility	Symptomatic; small sample size	Early clinical / Phase II	[84–88]
Ex vivo gene-corrected keratinocyte grafts (LAMB3 or COL7A1)	Autologous genetically corrected cells; restores structural protein expression	DEB/JEB; durable wound closure; potential phenotype correction	Complex manufacturing; limited availability; invasive	FDA-approved (Zevaskyn); others investigational	[89–94]
Cultured skin allografts (Apligraf, OrCel)	Epidermal/dermal constructs; growth-supportive matrices; may include autologous or allogeneic cells	Supports acute/chronic wound healing; reduces donor site morbidity; surgical adjunct	Allogeneic components; limited durability; not curative; EB-specific approvals limited	Clinical / early translational	[23, 95–98]

Abbreviations: *EB* Epidermolysis bullosa, *DEB* dystrophic EB, *JEB* junctional EB, *RDEB* recessive dystrophic EB, *FDA* Food and Drug Administration, *HSV*-1 herpes simplex virus type 1, *COL*7 type VII collagen, *ECM* extracellular matrix, *CCS* composite cultured skin, *B-VEC*; Vyjuvek Beremagene-geperpavec, and *PEG* polyethylene glycol

Chronic wounds are major therapeutic challenge of EB, and conventional dressings, while essential, are often limited by poor adherence, inadequate coverage, and insufficient modulation of the wound microenvironment [99, 100]. Next-generation wound dressings aim to move beyond passive protection, integrating bioactive, regenerative, antimicrobial, and smart-responsive properties to actively support tissue repair and improve patient outcomes [99]. These technologies leverage advances in biomaterials science, tissue engineering, and drug delivery to address the complex challenges posed by fragile, non-healing EB skin.

#### Ergonomic wound dressings

A notable recent development in EB wound care currently undergoing early preclinical testing is the creation of a seamless knitted dressing glove, produced through an iterative co-design process with individuals living with recessive dystrophic EB, their carers, clinicians and textile engineers [44]. Unlike conventional patch-based hand dressings that require multiple layers, adhesive tapes and secondary bandages which often result in increasing friction, maceration, heat build-up and painful removal, the dressing glove is engineered to conform anatomically to the hand and web spaces, providing consistent separation of digits while maintaining breathability and comfort. The material is a viscose–elastane blend finished with a low-adherence silicone coating and an antimicrobial treatment to reduce frictional trauma, suppress bacterial growth and prevent odour. The glove’s seamless structure enhances wearability and reduces the risk of blister formation caused by pressure points or wrinkles, while its moderate absorbency enables efficient uptake of blister fluid and rapid drying, thereby limiting moisture accumulation and maceration. Importantly, the knitted fabric slackens when wet, enabling atraumatic removal during soaking thus addressing one of the most distressing aspects of EB hand care. Surrogate and bench testing demonstrated improved conformability, tactile comfort and compatibility with web-spacer gloves used to maintain finger separation post-surgery. This patient-driven design represents a shift towards functional, garment-based dressings that prioritise usability, autonomy and reduction of dressing-change burden, and exemplifies how integrating textile technology, material science and co-design principles can generate next-generation EB-specific wound dressings with enhanced clinical relevance.

In contrast, Biobrane, a silicone–nylon mesh dressing incorporating porcine collagen now commercially available, has primarily been evaluated in the context of acute, post-surgical wound management in EB, particularly following release of hand contractures and pseudosyndactyly [45]. In six cases, it was applied as a glove-like dressing, allowing fast, easy application, promoting epithelialisation, and reducing the risk of iatrogenic trauma during dressing changes. Biobrane also facilitated exposure of healing skin, prevented maceration, and enabled the removal of additional protective layers. Healing time and the number of procedures required were influenced by the severity of hand deformities rather than the dressing itself, with total recovery not shortened below four weeks. while Biobrane may be a valuable adjunct for short-term, post-operative wound coverage in EB, its role differs from that of textile-based dressing gloves, which are designed to support long-term, low-trauma daily wound care and functional preservation.

#### Hydrogel and extracellular matrix -mimetic dressings

Hydrogels represent a promising class of biomaterials for EB wound management, with several hydrogel-based dressings already in routine commercial and clinical use, due to high water content and favourable physical properties that closely mimic the extracellular matrix. By maintaining a moist wound environment and providing gentle mechanical protection, hydrogel and other extracellular matrix -mimetic dressings—engineered to replicate the biochemical and structural cues of native dermal extracellular matrix —support the balanced exchange of gases, nutrients, and moisture while promoting cellular migration [46, 47]. Hydrogel advanced materials also provide a platform for integrating nanoscale therapeutics and responsive drug delivery systems, bridging material science and molecular therapy. Chitosan-based hydrogels have shown particular efficacy in enhancing wound healing by promoting cell proliferation, reducing inflammation, and exhibiting antimicrobial activity [48], and can be tailored to meet the specific requirements of fragile EB skin [49]. Alginate and alginate-hybrid materials have likewise been explored for biocompatibility, ability to form gels under physiological conditions, and capacity to deliver antimicrobial agents in a sustained manner [50]. In addition to a passive protective role, hydrogels can also be engineered to incorporate growth factors, peptides, or small molecules, enabling localised therapeutic delivery to support re-epithelialisation and angiogenesis [51].

At the pre-clinical stage, a temperature-sensitive polyhexanide gel has been developed that solidifies at body temperature, providing a gentle interface for patients with superficial wounds [52]. It is liquid at room temperature but forms a gel at or above body temperature, reducing pain associated with dressing changes and improving outcomes such as staining, leakage, and odour compared with traditional silver sulfadiazine dressings.

Another promising dressing recently described in in vitro studies involves a gelling dendritic hydrogel with engineered three-stage bonding, enabling strong initial adhesion followed by on-demand dissolution [53]. This system is based on a lysine-centred dendron and a PEG-based crosslinker that rapidly polymerise to form a conformable hydrogel within minutes of application. In vivo burn-wound studies show that this dressing provides effective haemostasis, prevents bacterial infection, and maintains a moist wound environment conducive to healing. A key breakthrough is its ability to be dissolved atraumatically through a controlled thiol–thioester exchange reaction, allowing clinicians to remove the dressing without disturbing fragile neo-epithelium—a feature of particular relevance to EB, where even minimal mechanical trauma can exacerbate blistering and impede healing. extracellular matrix -mimetic scaffolds further complement these approaches by providing architectural guidance that resembles native dermal structure, encouraging fibroblast and keratinocyte infiltration and supporting organised tissue regeneration [101].

Despite these advances, maintenance of a moist wound environment must be carefully balanced against the risk of periwound maceration, a frequent complication in EB that can facilitate wound expansion and delayed healing [102, 103]. Traditional occlusive foams and contact layers often provide limited control over exudate distribution, increasing the likelihood of moisture accumulation at wound margins [101, 102]. In contrast, contemporary hydrogel systems are increasingly engineered with tunable swelling properties, optimised crosslinking density, and semi-permeable architectures that permit controlled fluid handling while preserving hydration at the wound bed [45–47]. These design features enable more precise regulation of the wound microenvironment, reducing maceration risk while retaining the therapeutic benefits of moisture-retentive dressings [45, 46].

#### Nanotechnology-enabled dressings

Nanotechnology plays a pivotal role in the evolution of advanced wound dressings for EB, enabling the development of drug-loaded systems that can provide targeted therapeutic effects. Nanofibrous mats made from polymers such as poly(ε-caprolactone) have been shown to effectively encapsulate antibiotics and release them in a controlled manner, enhancing topical antimicrobial efficacy and reducing the frequency of dressing changes [54, 55]. Beyond infection control, nanotechnology-based platforms are increasingly being explored at the preclinical stage in EB-associated complications, including the management and treatment of aggressive squamous cell carcinoma arising from chronic non-healing wounds, reflecting broader interest in nanoparticle-mediated therapeutic delivery in this patient population [104]. However, given the extensive surface area of open wounds in EB, the safety of nanoparticle-based systems warrants careful consideration. Studies investigating systemic absorption and nanoparticle-associated toxicity have reported mixed outcomes, highlighting the need for rigorous evaluation of dose, particle composition, and exposure duration when translating these technologies towards clinical application [105–107]. Furthermore, the integration of self-responding mechanisms into wound dressings can significantly enhance patient outcomes. Self-responsive mats that change colour or release drugs in response to pH variations or the presence of infection can provide real-time indications of wound status and consequently reduce the overuse of antibiotics [56, 57].

### Topical bioactive and molecular therapies

Bioactive and molecular therapies represent an important component of the evolving therapeutic landscape in EB, encompassing both investigational strategies and agents that have achieved regulatory approval. These approaches aim to modulate disease-relevant molecular pathways, enhance wound healing, and improve dermal–epidermal stability through targeted biological or protein-based mechanisms. While several modalities remain in early or mid-stage clinical development, the approval of selected therapies provides proof-of-concept that molecularly targeted interventions can deliver clinically meaningful benefit in EB.

#### Topical gene therapy

Vyjuvek (Beremagene-geperpavec, B-VEC) is the first FDA-approved topical gene therapy for dystrophic-EB, delivered as a gel directly to wounds and targeting the underlying defect in type VII collagen (COL7) production [108]). Using a non-replicative herpes simplex virus type 1 vector, B-VEC introduces the COL7 gene to restore anchoring fibrils and accelerate re-epithelialisation. In a phase 3 randomized controlled trial involving 31 patients with DEB, weekly treatment of size-matched wounds with B-VEC significantly improved complete wound healing at both 3 and 6 months compared with placebo, with only mild adverse effects such as pruritus and chills reported [63, 64]. However, interpretation of these findings is constrained by the small cohort size, the predominance of recessive dystrophic-EB, the relatively short trial duration, the intrapatient study design, and the absence of histological confirmation of COL7 restoration. Importantly, while it is demonstrated that wound closure has been achieved, the long term durability of benefit and effects on wound recurrence remain incompletely characterised.

B-VEC can be administered in-office or at home, and its topical gel format allows for flexible application to selected wound areas based on patient age, wound size, and location [65]. Further, practical considerations include careful cleansing of wounds, gentle handling to avoid trauma, use of hydrophobic layers to optimise absorption and retention of the therapy. Caution must be exercised when cleaning the wound as improper cleansing can lead to treatment deactivation. There remains a theoretical risk of immune responses directed against the HSV-1 vector or the novel C7 produced as a result of the therapy. Particularly, with repeated long term exposure, although clinically significant immunogenicity has not been clearly demonstrated to date [28]. In clinical trials, treatment was associated with seroconversion to HSV-1 or type VII collagen in a proportion of patients; however, these immune responses were not correlated with increased adverse events, loss of efficacy, or clinically meaningful safety signals over the duration of follow-up [63, 64]. Weekly dosing ensures sustained COL7 expression and repeat applications may be targeted to wounds that are slow to heal or re-open. This flexible, patient-centric approach addresses both the molecular defect and the practical burden of wound care in dystrophic-EB. Unlike conventional supportive care, Vyjuvek addresses the root cause of dystrophic-EB at a molecular level, offering potential long-term benefits and reducing the frequency of hospital interventions. Vyjuvek presents a major cost burden, with an estimated annual price of around $300,000 per patient requiring lifelong weekly treatment, making it one of the most expensive gene therapies currently in use [66]. While further studies are needed, this therapy represents a major advance in disease-modifying treatments for dystrophic-EB, complementing ongoing efforts to improve wound management and patient quality of life.

#### Symptom-targeted therapies

INM-755 is a topical cannabinol cream under investigation for treating symptoms and healing wounds in patients with EB. The Phase II study (NCT04908215) enrolled patients across inherited EB subtypes and used a within-patient, double-blind design comparing INM-755 with control cream applied to matched areas over a short treatment period. Sponsor communications report signals for pruritus improvement and acceptable tolerability, with no serious drug-related adverse events and no withdrawals attributed to treatment [69]. No formal mechanistic studies in EB have been reported, however, preclinical evidence suggests that cannabinoids may exert antipruritic effects through modulation of neuronal activation, afferent signalling, and local activity on keratinocytes and mast cells, providing a potential rationale for symptom relief [70–73]. Efficacy outcomes were based on sponsor-reported data and congress materials rather than peer-reviewed outputs; therefore, conclusions about clinical benefit remain preliminary and limited by small sample size and short duration [74].

Topical ropivacaine has been studied as a local analgesic option to reduce procedural pain during wound care and dressing changes in EB. Ropivacaine is a long-acting amide local anaesthetic that reversibly inhibits the nociceptive influx of sodium ions in nerve fibres by blocking propagation of action potentials [75]. It also reduces the activity of the Transient Receptor Potential Vanilloid 1 channel, thus potentially contributing to control of neuropathic pain. Topical ropivacaine is known to be well tolerated [76]. A Phase II study (NCT03730584) to determine whether topical application of Ropivacaine is effective for treating refractory pain during dressing changes and improve quality of life of patients (newborn, child, adolescent or adults under 21) suffering from hereditary EB has been registered and is due to complete in 2028. Additionally, a case series in the British Journal of Dermatology describes use of topical ropivacaine in children and young adults with hereditary EB and reports rapid pain relief with good tolerance, though evidence remains limited, and the approach is symptomatic rather than disease-modifying [77].

#### Pathway- or mechanism-targeted interventions

The FDA approved Filsuvez, a topical birch bark triterpene gel, for the treatment of junctional EB and DEB in December 2023 [66]. Birch bark triterpenes have been shown to accelerate wound healing through upregulation of pro-inflammatory factors and increased keratinocyte migration [67]. In a phase 3 trial involving 223 EB patients, Filsuvez improved the rate of early complete wound closure compared with placebo; however, higher rates of severe adverse events were observed in the treatment group [68]. The therapeutic benefit was statistically significant only in patients with recessive DEB, with no clear efficacy in dominant DEB or junctional EB, and long-term outcomes remain unclear due to short follow-up. Additional limitations included incomplete follow-up, lack of improvement in secondary endpoints such as pain and disease severity scores, and a complex regulatory pathway requiring additional confirmatory data. Importantly, while Filsuvez may accelerate wound healing, it does not address the underlying genetic defect in EB, and wound recurrence remains likely, positioning it as a symptomatic rather than disease-modifying therapy.

Diacerein is a repurposed anthraquinone derivative with anti-inflammatory activity, including modulation of IL-1 signalling, explored as a targeted topical therapy for EB-simplex [109]. Early proof-of-concept work suggested reductions in blistering in severe EB-simplex treated with topical diacerein [78], and a subsequent Phase 2/3 placebo-controlled trial also reported clinically meaningful reductions in blister counts in treated episodes [79]. A later multicentred, randomized, vehicle-controlled trial (NCT03154333) evaluated diacerein 1% ointment in a broader EBS population. In the published report of this trial, the study did not meet the prespecified primary endpoint overall, although post-hoc subgroup analyses suggested a possible signal in more severe disease [80]. These results highlight challenges of heterogeneity and endpoint selection in EB-simplex topical therapy development.

BM-3103 (TolaSure) is a topical investigational therapy for severe EB-simplex, where there is a collapse of intermediate filament networks into intracellular aggregates under mechanical stress. BM-3103 is intended to target pathogenic keratin aggregation through an mTOR/autophagy-linked mechanism; preclinical studies identified BM-3103 as the first compound capable of stabilising intermediate filament networks in severe EB-simplex keratinocytes by promoting selective autophagic clearance of mutant keratin aggregates [81, 110]. This process is proposed to strengthen keratinocyte structural integrity while reducing inflammation, apoptosis, and blister formation. The Phase I/IIa TAMES study (NCT05062070), a double-blind, randomised, placebo-controlled trial, results have not been revealed at the time of this review. Interim biomarker and serial biopsy analyses from early participants have demonstrated improved keratinocyte organisation, enhanced cell–cell adhesion, and reduced keratin aggregates in treated lesional skin, supporting the proposed mechanism of action. However, peer-reviewed clinical efficacy data are not yet available, and definitive conclusions regarding clinical benefit await reporting of trial safety and efficacy endpoints.

Topical sirolimus, an mTOR inhibitor repurposed from systemic use, has been evaluated in plantar EBS in a Phase II trial (NCT03016715) [84]. Sirolimus as an oral tablet has well-established clinical use in preventing organ transplant rejection and in the treatment of rare diseases [85, 86]. In addition, Lee et al. described two adult patients with plantar EBS treated with sirolimus 2% ointment applied twice daily for 12 weeks, reporting reductions in blistering and plantar keratoderma, improved mobility, and decreased pain, with no treatment-related adverse events observed [87]. mTOR inhibition is proposed to attenuate keratinocyte hyperproliferation and inflammatory signalling, thereby improving epidermal stability and reducing susceptibility to mechanically induced injury [88].

Gentamicin, an aminoglycoside antibiotic, has been repurposed as a nonsense mutation read-through therapy for recessive dystrophic EB (RDEB) in patients with COL7A1 nonsense variants [82]. In a small pilot study, topical gentamicin applied to erosions (and intradermal gentamicin to intact skin) increased type VII collagen expression at the dermal-epidermal junction and was associated with improvements in treated areas, without reported induction of anti-type VII collagen autoantibodies [82]. Subsequent clinical experience also describes topical gentamicin use in EB caused by nonsense mutations, but evidence remains limited and larger studies are needed to define optimal dosing, durability, and clinically meaningful outcomes [83].

### Cell- and tissue-based interventions

Tissue engineering is rapidly advancing from basic research into clinically relevant and commercially available applications. Numerous skin substitutes have been developed in vitro, available as epidermal, dermal, or composite dermo-epidermal constructs, which may incorporate either cell-based or cell-free scaffolds. Building on these platforms, ex vivo gene and cell-based therapies represent a promising, though technically complex, strategy for treating EB, particularly in cases where topical or conventional interventions are insufficient. These approaches involve the genetic correction of patient-derived or donor cells outside the body, followed by transplantation to restore skin integrity and function.

#### Ex vivo gene-modified grafts

Autologous keratinocyte gene therapy (LAMB3-corrected grafts) was first demonstrated in junctional EB [89], where genetically corrected epidermal stem cells regenerated functional skin with durability exceeding five years, highlighting the potential for long-term disease correction [90]. Autologous COL7A1-corrected keratinocyte sheets (Zevaskyn, Abeona Therapeutics) were developed for recessive dystrophic EB and involve retroviral correction of patient keratinocytes followed by epidermal grafting [91]. Recently FDA-approved as a single-dose therapy, Zevaskyn showed significant wound closure and pain reduction, with long-term benefit observed in early-phase trials [92, 111], . However, trial limitations including small sample size, open-label design, and a primary endpoint of only 50% wound healing at 24 weeks limit the generalisability and interpretation of the results [93]. Ex vivo gene-corrected fibroblast therapy (Castle Creek Biosciences) uses lentiviral modified autologous fibroblasts delivered by intradermal injection. While shown to be safe in early studies, the absence of newly formed anchoring fibrils suggests limited durability and the need for repeat dosing [94]. Limitations include high cost, complex manufacturing, and the need for repeated treatments, which may hinder long-term practicality and accessibility. Collectively, ex vivo therapies offer the possibility of durable or single-administration benefit and may address aspects of EB beyond the skin, but high cost, logistical burden, and safety concerns currently limit widespread use compared with emerging topical and in vivo approaches.

#### Cultured skin allografts

Cultured skin allografts offer a promising adjunct or partial alternative to autografts in EB, particularly for surgical management of chronic wounds or hand deformities such as syndactyly and flexion contractures. It is important to distinguish cultured skin allografts from autologous keratinocyte grafts, as allografts provide temporary biological wound coverage and paracrine support but do not permanently engraft, with donor cells typically lost within weeks. In a series of operations on children with Recessive Dystrophic -EB, improved composite cultured skin (CCS) allografts were applied to donor sites and areas adjacent to autografts [23]. CCS-treated sites demonstrated good to excellent morphological and functional outcomes, reduced the size of autografts required, prolonged the interval to contracture recurrence, and provided superior donor sites for subsequent procedures. These findings highlight the potential of cultured skin allografts to enhance postoperative healing while minimising donor site morbidity in EB patients.

Tissue-engineered skin (Apligraf; Organogenesis Inc.) has been evaluated for wound healing in patients with various types of EB. In an open-label study of 15 patients, up to two wounds per patient were treated across three visits (day 1, week 6, and week 12) and assessed 7 days and 6 weeks after each treatment [95]. Of 69 acute wounds treated, 79% were healed by day 7, and healing rates remained high at later assessments (up to 82% at 6 weeks). Nine chronic wounds were also treated, with partial healing observed. No adverse events, infections, or graft rejections were reported. Patients reported faster, less painful healing and improved quality of life compared to conventional dressings. This study suggests that tissue-engineered skin is a safe and effective adjunct for EB wound care, particularly for acute wounds.

OrCel, developed by Forticell Bioscience, is an FDA approved resorbable, biocompatible cultured skin substitute containing fibroblasts and keratinocytes [112]. The fibroblasts are embedded in a porous collagen matrix, while keratinocytes form a nonporous epidermal layer [96]. These cells secrete a range of cytokines and growth factors (e.g., FGF-1, NGF, GM-CSF, IL-1α/β, KGF-1, VEGF) that condition the wound bed and support healing, particularly for autograft donor sites in burns and dystrophic EB. OrCel provides a favorable environment for host cell migration and wound repair but lacks dermal appendages. Allogeneic cells are cleared within three weeks, reducing the risk of long-term immune complications. However, no statistically significant difference was observed with respect to the time to wound healing after three treatments when OrCel was compared with its collagen sponge component alone or with standard care [97].

### Future perspectives

The therapeutic landscape for EB is undergoing a period of evolution, driven by advances in biomaterials science, molecular therapeutics, and tissue engineering. While gene- and cell-based strategies offer the prospect of durable disease modification, widespread clinical implementation remains constrained by cost, manufacturing complexity, regulatory hurdles, and the need for highly specialised infrastructure. Consequently, advanced wound dressings and topical or locally applied therapies are likely to remain central to EB management in the foreseeable future, serving both as primary interventions and as critical adjuncts to emerging disease-modifying approaches.

Future progress in EB wound care will depend on improved integration between material-based technologies and molecular therapies. Advanced dressings that provide mechanical protection, moisture balance, and antimicrobial control increasingly function as active therapeutic platforms rather than passive barriers. Incorporating bioactive agents, nanoparticles, or responsive drug delivery systems within these dressings may enable sustained, localised modulation of inflammation, infection, and re-epithelialisation while minimising trauma during application and removal. Nanoparticle-based systems are particularly well suited to this role, as the small size of particles (typically ≤ 100 nm) confers advantages in chronic wound environments, including enhanced drug solubility, improved bioavailability, and prolonged local retention while limiting systemic exposure [59]. In addition, nanoparticles can protect encapsulated drugs, peptides, and proteins from enzymatic degradation and premature inactivation—an important consideration for bioactive molecules with short in vivo half-lives, such as growth factors or anti-inflammatory peptides relevant to EB wound healing [60–62].

Liposome-based nanoparticle formulations may be particularly attractive in this context and are currently being investigated for dermatologic use, including the topical delivery of chemotherapeutic and immunomodulatory agents for skin cancers and inflammatory skin diseases, where these systems provide a sustained release of drug, enhanced local drug retention, penetration, and tolerability [60, 113]. The capacity for cellular interaction and rapid internalisation via endocytic pathways allows nanoparticle systems to also enable intracellular targeting of inflammatory or reparative pathways within keratinocytes, fibroblasts, or immune cells, which are central to EB wound pathophysiology [59, 114]. Incorporated into soft wound dressings, hydrogels, or tissue-engineered constructs, these platforms offer a minimally invasive and adaptable strategy that aligns closely with the clinical priorities of EB care. These combination strategies align closely with the clinical realities of EB, where frequent dressing changes, pain, and cumulative skin damage impose a substantial burden on patients and caregivers.

Smart or responsive dressings represent a next-generation approach to wound care in EB. These dressings are designed to actively respond to the wound environment, releasing therapeutic agents such as antimicrobials, growth factors, or anti-inflammatory compounds in response to stimuli like pH [115], or enzymatic activity [116]—parameters often dysregulated in chronic EB wounds due to persistent inflammation, infection, and tissue breakdown [117, 118]. By adapting dynamically to changing wound conditions, smart dressings can optimise healing, reduce infection risk, and minimise the frequency of painful dressing changes. Integration with sensors or hydrogel matrices can provide real-time feedback on wound status, offering opportunities for personalised, targeted therapy while protecting fragile EB skin [119]. These technologies, still largely in preclinical development and tested across a range of in vitro cell and animal models, hold significant potential to complement existing and emerging molecular therapies in EB. Topical molecular therapies, including gene delivery, pathway-targeted agents, and repurposed drugs, have demonstrated proof-of-concept that locally applied treatments can yield clinically meaningful benefit in EB. However, future studies must prioritise durability of response, long-term safety, and patient-relevant endpoints beyond short-term wound closure. This includes assessment of sustained wound stability, recurrence rates, pain reduction, infection burden, and functional outcomes that better reflect daily disease impact. Careful stratification by EB subtype, disease severity, and molecular mechanism will be essential to identify patients most likely to benefit from specific interventions. In parallel, the distinction between symptomatic relief and true disease modification must be clearly defined to guide clinical decision-making and regulatory evaluation. In addition to current pipelines, systematic investigation of repurposed anti-inflammatory drugs targeting IL-1, TNF-α, or mTOR signalling, antioxidant compounds such as polyphenols, flavonoids, and thiol-based redox modulators, as well as naturally derived bioactive molecules with demonstrated efficacy in other dermatological or chronic wound-healing contexts, may offer additional avenues for topical intervention in EB [120–122]. These strategies are particularly relevant given the contribution of oxidative stress and persistent inflammation to impaired healing and tissue fragility, and may enable more rapid clinical translation where safety profiles or mechanistic rationale are already established.

Cell- and tissue-based interventions remain among the most promising yet challenging approaches. Ex vivo gene-corrected grafts and cultured skin substitutes have shown the capacity for durable regeneration and improved wound healing, but scalability and accessibility are limited. Advances in 3D bioprinting, bioengineered scaffolds, and hybrid constructs incorporating autologous or gene-corrected cells may help bridge the gap between experimental success and routine clinical use [123]. Through precise, layer-by-layer deposition of multiple cell types within tailored extracellular matrix environments, 3D bioprinting offers the potential to restore both structural integrity and barrier function while promoting wound healing [124]. Recent advances have focused on the development of ‘pro-regenerative’ grafts incorporating cell-instructive cues, including growth factors, antimicrobial agents, bioactive nanoparticles, and cell-binding peptides, to actively modulate the wound microenvironment and support durable regeneration [125–127]. Importantly, 3D bioprinting may also serve as a platform for the delivery of gene-edited or gene-corrected cells, offering a route toward phenotype correction in recessive dystrophic EB, as well as enabling disease modelling of EB-associated squamous cell carcinoma [127]. Despite this promise, significant challenges remain, including optimisation of bioinks for print fidelity and biological function, bridging the gap between in vitro constructs and in vivo healing responses, and overcoming immunological and translational barriers [123]. Regardless, combining these approaches with advanced dressings —such as nanoparticle-mediated delivery of growth factors or responsive dressings that adapt to local wound conditions—or local molecular therapies may further enhance graft integration, reduce recurrence, and improve long-term functional outcomes.

Across all therapeutic modalities, future EB research will benefit from more standardised outcome measures, longer follow-up periods, and incorporation of mechanistic biomarkers alongside clinical endpoints. Patient-centred design, including co-development of therapies and dressings with individuals living with EB, should remain a priority to ensure that innovations translate into meaningful improvements in daily care, autonomy, and quality of life.

## Conclusion

The therapeutic landscape for EB is evolving, driven by advances in biomaterials, molecular therapies, and tissue engineering. While gene- and cell-based strategies hold promise for durable disease modification, practical constraints limit immediate accessibility. Consequently, advanced wound dressings and topical interventions remain central to EB management, providing both primary care and adjunctive support to emerging therapies. Integration of bioactive, regenerative, and smart-responsive features, including nanoparticle delivery systems, enables localised modulation of inflammation, infection, and tissue repair while minimising trauma to fragile skin. Topical molecular therapies, repurposed or novel drugs, and naturally derived bioactive molecules further expand the therapeutic repertoire, offering opportunities for rapid translation based on established safety and mechanistic rationale. Tissue- and cell-based approaches, including ex vivo gene-corrected grafts, cultured skin substitutes, and 3D-bioprinted constructs, provide potential for durable regeneration, particularly when combined with advanced dressing technologies. Future progress will depend on the adoption of harmonised and clinically meaningful endpoints. Recent interventional studies in EB have highlighted limitations associated with traditional efficacy measures, particularly binary endpoints such as complete wound closure. In a chronic and recurrent disease characterised by ongoing blister formation and variable wound chronicity, such endpoints may inadequately capture partial but clinically relevant treatment effects. Increasing evidence supports the inclusion of alternative outcome measures, such as changes in wound burden and persistence, reduction in itch and pain, frequency of secondary infection, and improvements in functional outcomes and patient-reported quality of life. Integration of these patient-centred endpoints, alongside mechanistic biomarkers of inflammation and tissue repair, will be critical for accurately assessing therapeutic benefit and guiding the clinical translation of emerging EB treatments.

## Data Availability

No datasets were generated or analysed during the current study.

## Declarations

### Use of generative AI

ChatGPT (GPT-5 mini) was used to assist with language editing and improving clarity of the manuscript. All scientific content, interpretations, and conclusions remain solely those of the authors.
